# A Spherical Hybrid Triboelectric Nanogenerator for Enhanced Water Wave Energy Harvesting

**DOI:** 10.3390/mi9110598

**Published:** 2018-11-15

**Authors:** Kwangseok Lee, Jeong-won Lee, Kihwan Kim, Donghyeon Yoo, Dong Sung Kim, Woonbong Hwang, Insang Song, Jae-Yoon Sim

**Affiliations:** 1 Department of Mechanical Engineering, Pohang University of Science and Technology (POSTECH), Pohang 37673, Korea; shepherd@postech.ac.kr (K.L.); aaron@postech.ac.kr (J.-w.L.); kihwan@postech.ac.kr (K.K.); ta2two@postech.ac.kr (D.Y.); smkds@postech.ac.kr (D.S.K.); 2 Agency for Defense Development (ADD), Daejeon 34186, Korea; energysong@add.re.kr; 3 Department of Electrical Engineering, Pohang University of Science and Technology (POSTECH), Pohang 37673, Korea; jysim@postech.ac.kr

**Keywords:** triboelectric nanogenerator, energy harvesting, hybrid energy, water wave energy

## Abstract

Water waves are a continuously generated renewable source of energy. However, their random motion and low frequency pose significant challenges for harvesting their energy. Herein, we propose a spherical hybrid triboelectric nanogenerator (SH-TENG) that efficiently harvests the energy of low frequency, random water waves. The SH-TENG converts the kinetic energy of the water wave into solid–solid and solid–liquid triboelectric energy simultaneously using a single electrode. The electrical output of the SH-TENG for six degrees of freedom of motion in water was investigated. Further, in order to demonstrate hybrid energy harvesting from multiple energy sources using a single electrode on the SH-TENG, the charging performance of a capacitor was evaluated. The experimental results indicate that SH-TENGs have great potential for use in self-powered environmental monitoring systems that monitor factors such as water temperature, water wave height, and pollution levels in oceans.

## 1. Introduction

The demand for energy has steadily increased since the development of the steam engine. In particular, in pursuit of convenience and advancement, the rapid growth and development of industries has prompted humans to explore new energy resources to meet their increasing energy demands. However, considering the need for alternative sources of energy owing to the non-renewable nature of fossil fuels, increasing environmental pollution due to the use of these traditional sources of energy, and consequent global warming, energy harvesting from natural sources (renewable energy) has become a hot topic in the field of energy technology [1,2,3,4,5,6,7,8,9,10,11]. Water waves are a renewable energy source, and water wave energy is continuously generated in oceans all around the world. It is theoretically estimated that 2 TW of energy per year could potentially be generated from water waves, which is more than our current and near-future energy requirements [12]. Although several previous studies have proposed various methods of harvesting water wave energy, the methods proposed in most of those studies are inefficient, complicated, and expensive because they typically involve converting wave energy into translational or rotational energy to operate an electromagnetic generator (EMG) [13,14,15]. In addition, the random motion and low frequency of water waves are significant hurdles in the generation of power using EMGs [16,17,18].

Triboelectric nanogenerators (TENGs) can overcome the abovementioned drawbacks as TENGs convert the mechanical energy of water waves into electricity based on a combination of the contact electrification and electrostatic induction phenomena [19,20]. The advantages of energy generation systems developed using TENGs include simple design, low weight, low cost, and high efficiency [19,21,22,23,24,25,26,27,28,29,30,31,32,33,34,35,36,37,38,39,40,41]. In addition, TENGs can generate energy from solid–liquid as well as solid–solid triboelectrification [42,43]. Because of these advantages, several types of TENGs have been proposed for harvesting water wave energy [44,45,46,47]. For example, turbine-based TENGs that generate energy by converting water wave energy into translational or rotational energy have been proposed [48,49]. In addition, encapsulated designs, including those for movable objects, have been proposed for efficiently harvesting the complex motion of water waves [50,51,52,53]. Furthermore, to harvest water wave energy via water contact electrification, TENGs with hydrophobic surfaces have been proposed [42,54].

In this work, based on these previous studies on TENGs for water wave energy harvesting, we propose an improved TENG called the spherical hybrid triboelectric nanogenerator (SH-TENG). By harvesting energy from multiple sources through one electrode simultaneously, our proposed SH-TENG provides a simplified approach to previous hybrid TENG systems [54,55,56]. Moreover, the simple fabrication process used for the proposed SH-TENG easily facilitates mass production, which can contribute to the commercialization and scalability of the proposed SH-TENG. Thus, SH-TENGs have great potential for use in self-powered environmental monitoring systems that monitor factors such as water temperature, water wave height, and pollution levels in oceans.

## 2. Experimental Section

### 2.1. Materials and Methods

**Figure 1:**
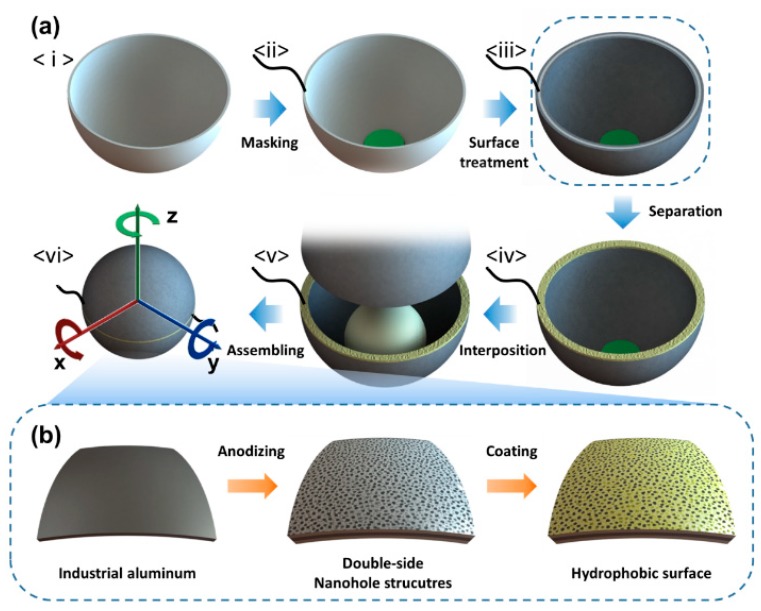
(**a**) Fabrication of the spherical hybrid triboelectric nanogenerator (SH-TENG). (**b**) Surface treatment of hydrophobic nanohole-structured anodic aluminum oxide.

The proposed SH-TENG was fabricated as follows. Industrial aluminum hemispherical shells with a diameter of 6 cm were immersed in 1 M NaOH for 5 min and then cleaned using deionized (D.I.) water. The cleaned hemispherical shells were masked in the center of the inner surface with a maskant (HTM-201, Woo Ju Chemical Co., Incheon-si, Korea) and a wire was connected to the edge of the shells. Then, the hemispherical shells were anodized in a 0.3 M oxalic acid solution at a constant voltage of 50 V for 100 min using a power supply (DRP-92001DUS, Digital Electronics Co., Incheon-si, Korea). The temperature of the solution was constantly maintained using a circulator (RW-0525G, Lab Companion Inc., Seoul-si, Korea) during the anodization process. Thereafter, the as-prepared shells were dipped in a mixture of n-hexane and heptadecafluoro-1,1,2,2,-tetrahydrodecyl trichlorosilane (C_10_H_4_Cl_3_F_17_Si, HDFS, Alfa Aesar, MA, USA), with a volumetric ratio of 1000:1, for 10 min and dried in an oven at 110 °C for 10 min. The coated hemispherical shells were then soaked in a solvent (C_10_HF_22_N, FC-40, Fluorinert^TM^, Saint Paul, MN, USA) with 0.1 wt% polytetrafluoroethylene ((C_2_F_4_)_n_, PTFE, Chemours^TM^, Wilmington, DE, USA), after which they were dried in an oven under vacuum at 60 °C for 24 h. The edge of the re-coated hemispherical shells was then covered with 1 mm thick polyamide adhesive to isolate each shell (Figure S1). Finally, a 3 cm diameter nylon ball was placed inside one shell and the two shells were joined together (Figure 1).

### 2.2. Characterization

The surface structure and cross section of the fabricated SH-TENG were observed using a field emission scanning electron microscope (SU66000, Hitachi, Tokyo, Japan). To evaluate the wettability of the SH-TENG surface, contact angles (CAs) were measured using a D.I. water droplet analysis tool (SmartDrop, FEMTOFAB, Seongnam-si, Korea). The D.I. water droplet was 5 µL.

### 2.3. Electrical Output Measurement of the Spherical Hybrid Triboelectric Nanogenerator (SH-TENG)

To analyze the electrical outputs from both the inner and outer surfaces of the SH-TENG, solid–solid and solid–liquid TENGs were fabricated in addition to the previously specified SH-TENG. More specifically, the solid–solid TENG was fabricated by covering the outer surface of the SH-TENG to prevent solid–liquid triboelectrification at the outer surface; in contrast, the solid–liquid TENG was fabricated by removing the nylon ball from the SH-TENG to prevent solid–solid triboelectrification at the inner surface. The electrical output of these three TENGs was then analyzed in a motion plan involving six degrees of freedom (6 DoFs), which were used to represent both a change in position and rotation along a perpendicular axis (Figure S2). The output voltage of the SH-TENG for translational and rotational motion was measured using a mixed signal-and-digital oscilloscope (DS1074Z, RIGOL, Beaverton, OR, USA). To vibrate the SH-TENG, it was mounted on an electrodynamic shaker (ET-140, Labworks Inc., Costa Mesa, CA, USA). In particular, for vertical vibrations, the SH-TENG was set to sink 2 cm in D.I. water and then rise with a frequency of 1 Hz. For horizontal vibrations, the SH-TENG was vibrated horizontally with 2 cm of it submerged in the water at a frequency of 1 Hz. Furthermore, to evaluate the output of the SH-TENG during rotational movement, with 2 cm of the SH-TENG submerged in D.I. water, it was rotated using a speed-control motor (E9I90PBH-TU, ExceM, Seoul-si, Korea); the rotational velocity was fixed at 60 rpm. In order to observe the electrical output of these TENGs during water wave motion, they were placed in a 100 cm × 70 cm × 20 cm bath. Then, a controllable water pump (EcoDrift 15.0, Aquamedic, Bissendorf, Germany) was operated to generate water waves. To rectify the electrical output, a rectifier (B40C800DM-E3/45, Vishay Semiconductors, Malvern, PA, USA) was used.

## 3. Results and Discussion

### 3.1. Design and Features of the SH-TENG

**Figure 2:**
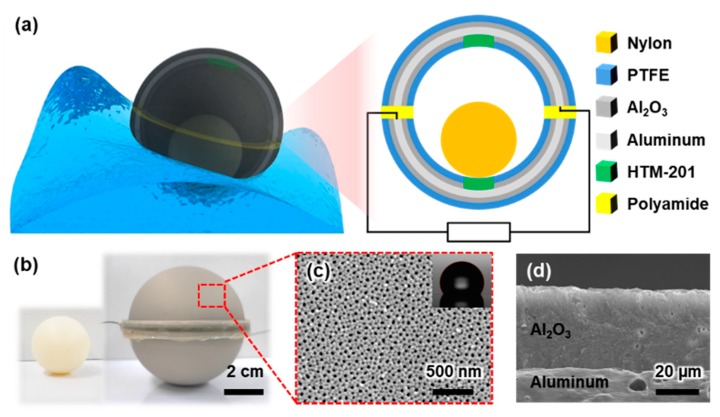
(**a**) Three-dimensional model and schematic diagram of the SH-TENG. (**b**) Photograph of the nylon ball and SH-TENG. (**c**) Scanning electron microscopy (SEM) image and contact angles (CAs) of the SH-TENG surface. (**d**) Cross-sectional SEM image of Al_2_O_3_.

A symmetric spherical design was adopted for our proposed SH-TENG in order to reduce loss of kinetic energy and enable flexible movement of the SH-TENG under the effect of random water waves. As shown in Figure 2a, all surfaces of the SH-TENG undergo water contact electrification because the hemispherical shell is made of aluminum, which acts as an electrode. In particular, the hemispherical shell is covered on both the inner and outer surfaces with a dual layer consisting of Al_2_O_3_ and PTFE. The Al_2_O_3_ layer serves as a dielectric layer that acts as an insulator to prevent leakage of charge (Figure 2d) [57]. PTFE, which has a strong natural tendency to be negatively charged in triboelectric series, is used as the friction layer in the SH-TENG because water tends to acquire a positive charge on triboelectrification [58]. Thus far, several TENGs have been manufactured that involve bonding or depositing these different layers separately [48,49,50,51,52,53,54,55,56]. However, in the case of the proposed SH-TENG, the bonding process is omitted because anodizing and self-assembled monolayer (SAM) coating methods are used to robustly develop Al_2_O_3_ and PTFE layers on the electrode without the need for adhesives; this is an advantage for mass production of the proposed SH-TENG. Furthermore, a nanohole structure is applied to the surface of the SH-TENG to enable the separation of water from the surface after contact, as well as to simultaneously maximize the frictional force experienced by the SH-TENG; the nanostructure is shown in Figure 2c [59]. In order to prevent the two shells from making electrical contact, a hot melt glue stick was used to cover the edge of the shells with a 1 mm thick polyamide layer. The high viscosity of the molten polyamide enabled it to be applied thickly to electrically isolate each shell. As previously mentioned, the hemispherical shells were partially masked with HTM-201 to prevent contact electrification in that area. Further, this masking enables the nylon ball to separate from the PTFE after making contact inside the hemispherical shell.

### 3.2. Operating Principle

**Figure 3:**
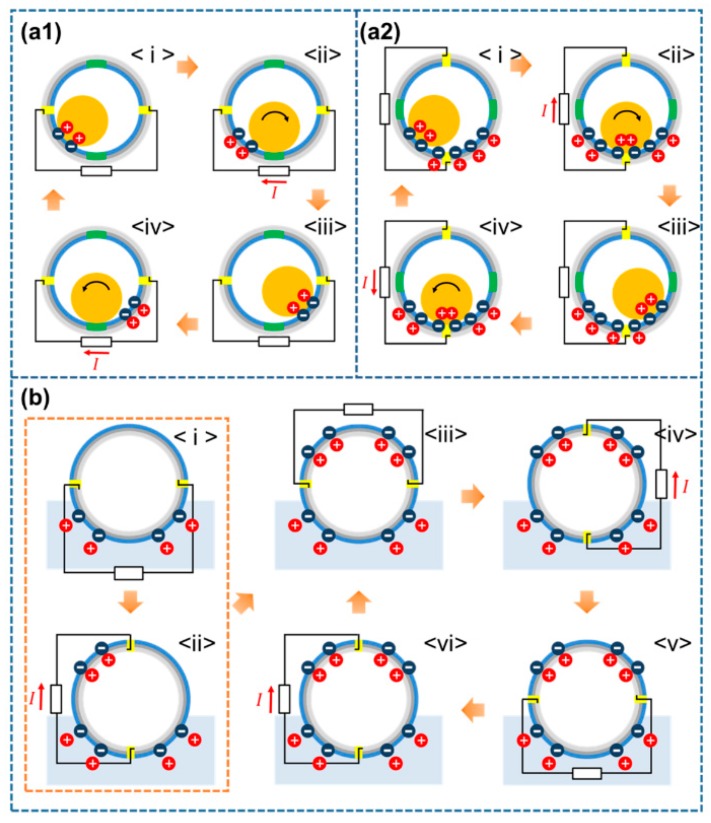
Schematic diagram representing the operating principle of the SH-TENG on its inner surface: (**a1**) single electrode triboelectric mode, and (**a2**) freestanding triboelectric mode. (**b**) Schematic diagram of the operating principle of the SH-TENG on its outer surface.

The proposed SH-TENG should be able to realize both solid–solid triboelectrification—which occurs as a result of contact separation between the PTFE surface and the nylon ball inside the SH-TENG—and solid–liquid triboelectrification, simultaneously. Figure 3(a1) shows a schematic diagram depicting triboelectrification between the hemispherical shell and the nylon ball. When the PTFE surface is in contact with the nylon ball, it has a negative charge, while the nylon ball has a positive charge. However, when the ball moves to the masked area owing to translational or rotational motion, electrostatic induction occurs because of the separation of the nylon ball from the PTFE. Then, when the nylon ball again comes into contact with the PTFE layer, contact electrification again occurs. Thus, as long as the SH-TENG is in motion, there will be repeated contact electrification and electrostatic induction. This series of processes can be considered as a single-electrode mode owing to contact separation of the electrified body for a single electrode [60]. Figure 3(a2) shows the triboelectric relationship between the nylon ball and two hemispherical shells. The electrons travel between the two hemispherical shells owing to the potential difference generated by contact electrification. Thus, a current is generated. This design, in which one dielectric object travels between two electrode layers, is similar to that of the freestanding triboelectric mode [61].

On the outer surface of the SH-TENG, solid–liquid triboelectrification between the PTFE layer and water leads to energy generation as well. When the SH-TENG is dipped in water, the outer surface of the SH-TENG gets negatively charged, while the surrounding water gets positively charged. This charged state of the SH-TENG is illustrated in Figure 3b <i>. As the SH-TENG is rotated by an external force, the immersed PTFE surface gets negatively charged. The PTFE in contact with water maintains an electrical balance with the water it is in contact with; however, the part of the PTFE that is not in contact with water experiences electrostatic induction in order to balance the electric charge generated on the immersed PTFE layer. This process causes charge transfer to occur between the two hemispherical shells. When the difference of the water contact area between the electrodes is ∆A, the electrical output is determined by the changing rate of ∆A (Equation (1)):

(1) $$V\;\propto \frac{d∆A}{dt}=\frac{d}{dt}\;\left(\mathrm{difference}\;\mathrm{of}\;\mathrm{area}\;\mathrm{in}\;\mathrm{contact}\;\mathrm{with}\;\mathrm{water}\;\mathrm{in}\;\mathrm{each}\;\mathrm{electrode}\right)$$

After all the surfaces have been charged once, the process indicated by Figure 3b <iii>a–<vi> is repeated.

### 3.3. Electrical Output of SH-TENG during 6 Degrees of Freedom (DoF) Motion

**Figure 4:**
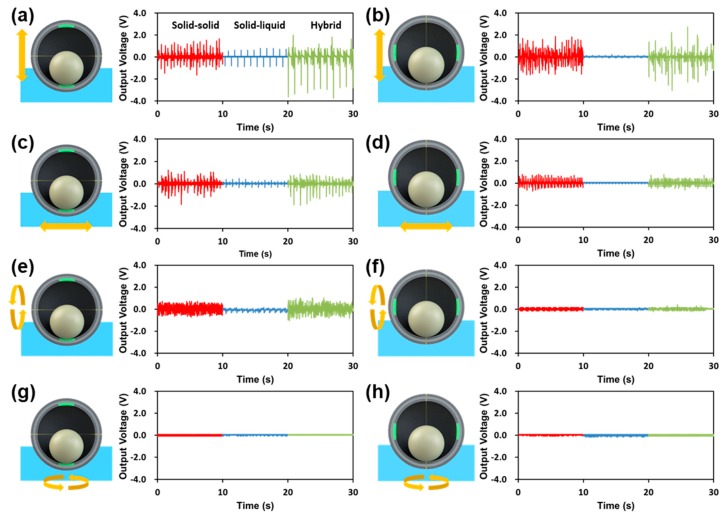
Schematic diagram and electrical output of the triboelectric nanogenerators (TENG): (**a**,**b**) during vertical vibration with respect to the (**a**) *z*- and (**b**) *x*-axes; (**c**,**d**) during horizontal vibration with respect to the (**c**) *x*- and (**d**) *z*-axes; (**e**,**f**) during rotation with respect to the (**e**) *x*-axis and (**f**) *z*-axis parallel to the water surface; (**g**,**h**) during rotation with respect to the (**g**) *z*-axis and (**h**) *x*-axis perpendicular to the water surface.

Solid–solid, solid–liquid, and hybrid electrical outputs were evaluated for 6 DoF motion (Figure 4). In order to effectively represent the different movements for the 6 DoF motion, an intrinsic coordinate system for the SH-TENG—depicted in Figure 1a <vi>—was used. Motion along the *x*- and *y*-axes of the SH-TENG is considered to be the same owing to the symmetric design of the SH-TENG. The electrical outputs from the translational and rotational motion about the *x*- and *z*-axes of the SH-TENG were measured.

Figure 4a–h includes graphs showing the electrical output voltage of the TENGs during translational or rotational motion. In particular, Figure 4a–d shows the electrical output during translational motion. Figure 4a,b shows the electrical output when the TENG is vertically immersed in D.I. water by 2 cm in the x- and z-axes directions of the SH-TENG. Because of the random motion of the nylon ball inside the SH-TENG, despite constant vibration, a relatively random electrical output is observed for solid–solid triboelectrification. In contrast, for solid–liquid triboelectrification, the electrical output is generated owing to the change in the area in contact with water between the two shells. In the case depicted in Figure 4a, water contact electrification of the lower shell leads to generation of an electrical output because the upper shell is not in contact with water. However, the low electrical output in the case shown in Figure 4b occurs because of the relative lack of difference in the water contact area between each electrode. Figure 4c,d shows graphs depicting the generated electrical output when horizontal vibration is applied to the SH-TENG while it is submerged 2 cm in the water. As in the case with vertical vibration, the randomly distributed energy output is attributed to the random motion of the nylon ball inside the SH-TENG. In particular, because of the small change in the water contact area during solid–liquid triboelectrification, its energy output is considerably lower.

For rotational motion, the energy output was measured with the SH-TENG immersed 2 cm in the water. The observed electrical outputs depend on the contact separation of the nylon ball as well as the change in the contact area between the two hemispherical shells and water. Figure 4e shows the electrical output when the SH-TENG is rotated with respect to the *x*-axis parallel to the water surface. For the other rotation, Figure 4f–h indicates that the electrical output is considerably lower because the nylon ball does not undergo contact separation motion with the PTFE; thus, its contact area with the water does not change.

### 3.4. Electrical Output of SH-TENG for Water Wave Motion

**Figure 5:**
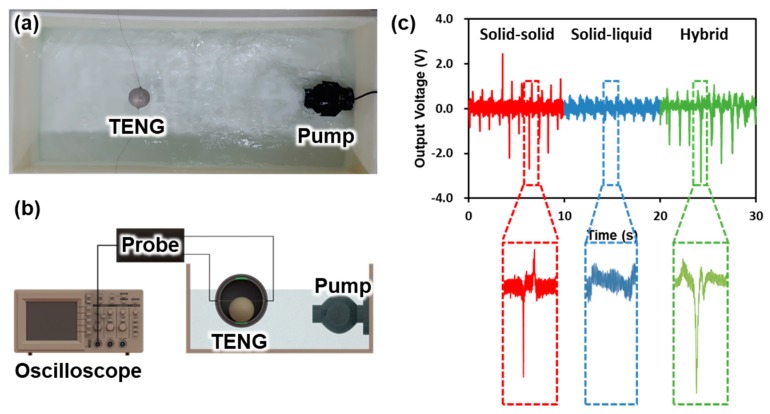
Experimental setup: (**a**) photograph and (**b**) schematic diagram of TENG for electrical output measurements in water wave motion. (**c**) Electrical output of TENG under wave motion with a frequency of 1 Hz.

Figure 5a,b illustrates the actual experimental setup and schematic diagram of the observed electrical output due to the water wave motion. The energy yield of the SH-TENG is evaluated for a 1 Hz water wave in the tank. As shown in Figure 5c, sporadic energy generation was observed per the contact separation phenomenon in the case of solid–solid triboelectrification; the maximum output voltage observed in this case was 2.7 V. In contrast, in the case of solid–liquid triboelectrification, it was confirmed that the energy generation occurred in a continuous form rather than in a sporadic form, because the electrical output is generated due to the change in the contact area between each electrode and the water; the maximum voltage observed in this case was 0.5 V. The combination of these two energies, i.e., energies of the sporadic and continuous form, are referred to as the hybrid form; the maximum peak voltage in this case was 3.3 V.

**Figure 6:**
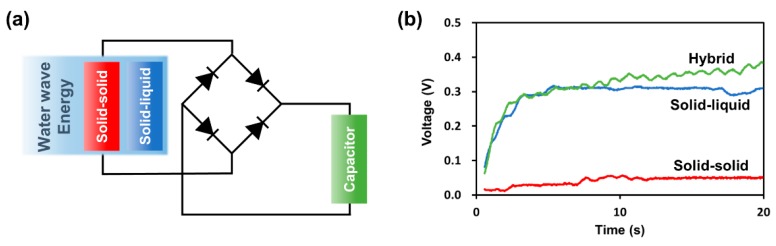
(**a**) Schematic diagram of the rectifying circuit. (**b**) Voltage of a 1 μF capacitor charged by the TENG for 20 s during water wave motion with a 1 Hz wave frequency.

In addition, to demonstrate whether the energy is harvested by the inner and outer surfaces simultaneously, the circuit shown in Figure 6a was constructed to charge a capacitor using the harvested water wave energy. Figure 6b shows the measured voltage of the 1 μF capacitor charged by the three types of TENGs for 20 s during water wave motion with a wave frequency of 1 Hz. The capacitor was charged to 0.08 V during solid–solid triboelectrification, 0.31 V during solid–liquid triboelectrification, and 0.39 V during hybrid triboelectrification. The maximum voltage during solid–solid triboelectrification is higher than that of solid–liquid triboelectrification as shown in Figure 5c; however, the output of the solid–liquid triboelectrification in terms of capacitor charging is higher than that of solid–solid triboelectrification in the case shown in Figure 6. It can be seen that there is a difference between the maximum peak level and the root mean square (RMS) level. Thus, our experimental results demonstrate that solid–solid triboelectrification and solid–liquid triboelectrification can be simultaneously harvested using one electrode. In addition, the charged energy of the 1 μF capacitor is shown in Figure S3. The charged energy can be derived from the charged voltage in the capacitor by the following equation (Equation (2)):

(2) $$E=\frac{1}{2}{CV}^{2}$$

where *E*, *C*, and *V* are the charged energy, size of the capacitance, and charged voltage, respectively. The charged energy had a similar tendency to the voltage in Figure 6b by Equation (2) (Figure S3).

## 4. Conclusions

SH-TENG, which flexibly responds to random water wave energy and harvests hybrid energy using a single electrode, was proposed in this paper for enhanced water wave energy harvesting. The electrical output was analyzed in 6 DoF motion for solid–solid, solid–liquid, and hybrid triboelectrification. In addition, using a capacitor, it was confirmed that solid–solid triboelectrification and solid–liquid triboelectrification are harvested simultaneously through the two sides of one electrode layer. These results indicate that it is possible to simplify the previously proposed hybrid TENG system to enable mass production of TENGs, which can contribute to the commercialization and scalability of blue (i.e., water wave) energy.

## Supplementary Materials

The following are available online at http://www.mdpi.com/2072-666X/9/11/598/s1.
