# Supplementary material for: A Spherical Hybrid Triboelectric Nanogenerator for Enhanced Water Wave Energy Harvesting
Source: Micromachines (Basel). 2018 Nov 15;9(11):598. doi: 10.3390/mi9110598 (PMC6266367; doi:10.3390/mi9110598)
Supplement: Supplementary file 1 [file micromachines-09-00598-s001.zip › Supplementary Information.pdf]

Supplementary Information

Spherical hybrid triboelectric nanogenerator for enhanced water wave energy harvesting

Kwangseok Lee [1], Jeong-won Lee [1], Kihwan Kim [1], Donghyeon Yoo [1], Insang Song [3], Dong Sung Kim [1], Jae-Yoon Sim [2] and Woonbong Hwang [1,*]

1 Department of Mechanical Engineering, Pohang University of Science and Technology (POSTECH), Pohang 37673, Republic of Korea; shepherd@postech.ac.kr (K.L.); aaron@postech.ac.kr (J.-W.L.); kihwan@postech.ac.kr (K.K.); ta2two@postech.ac.kr (D.Y.); smkds@postech.ac.kr (D.S.K.)

2 Department of Electrical Engineering, Pohang University of Science and Technology (POSTECH), Pohang 37673, Republic of Korea; jysim@postech.ac.kr (J.-Y. S.)

3 Agency for Defense Development (ADD), Daejeon 34186, Republic of Korea; energysong@add.re.kr (I.S.)

* Correspondence: whwang@postech.ac.kr (W.H.); Tel.: +82-54-279-2174

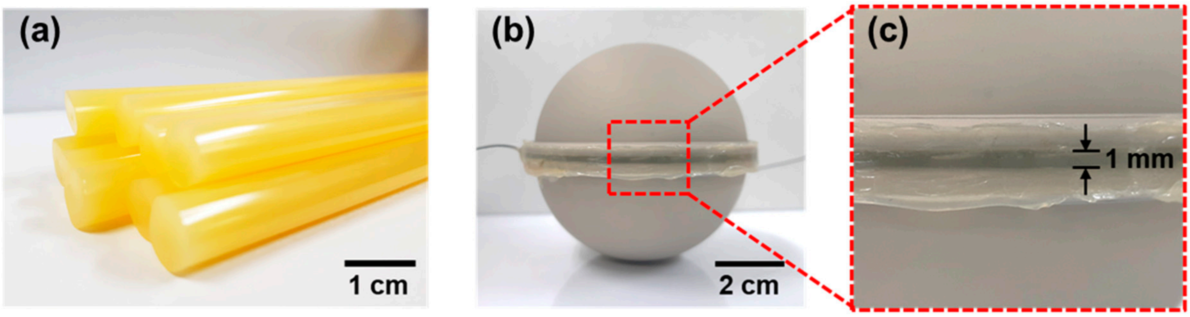

Figure S1 (a) Polyamide hot melt glue stick (b) Picture of the hemisphere shell with polyamide adhesive on the edge (c) Thickness of polyamide adhesive

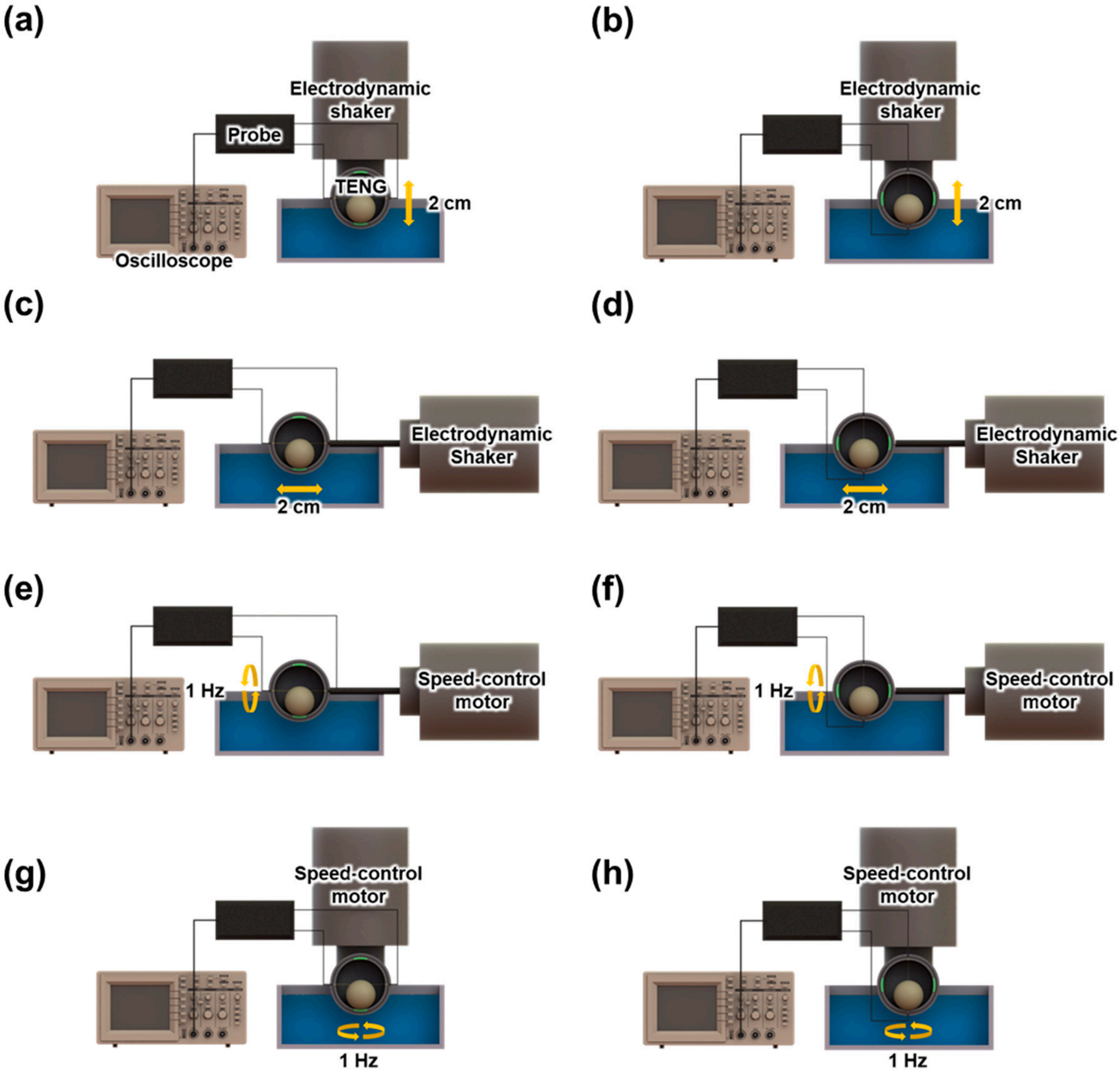

Figure S2 Schematic diagram of the experiments: (a,b) during vertical vibration with respect to the (a) z and (b) x axes; (c,d) during horizontal vibration with respect to the (c) x and (b) z axes; (e,f) during rotation with respect to the (e) x axis and (f) z axis parallel to the water surface; (g,h) during rotation with respect to the (g) z axis and (h) x axis perpendicular to the water surface.

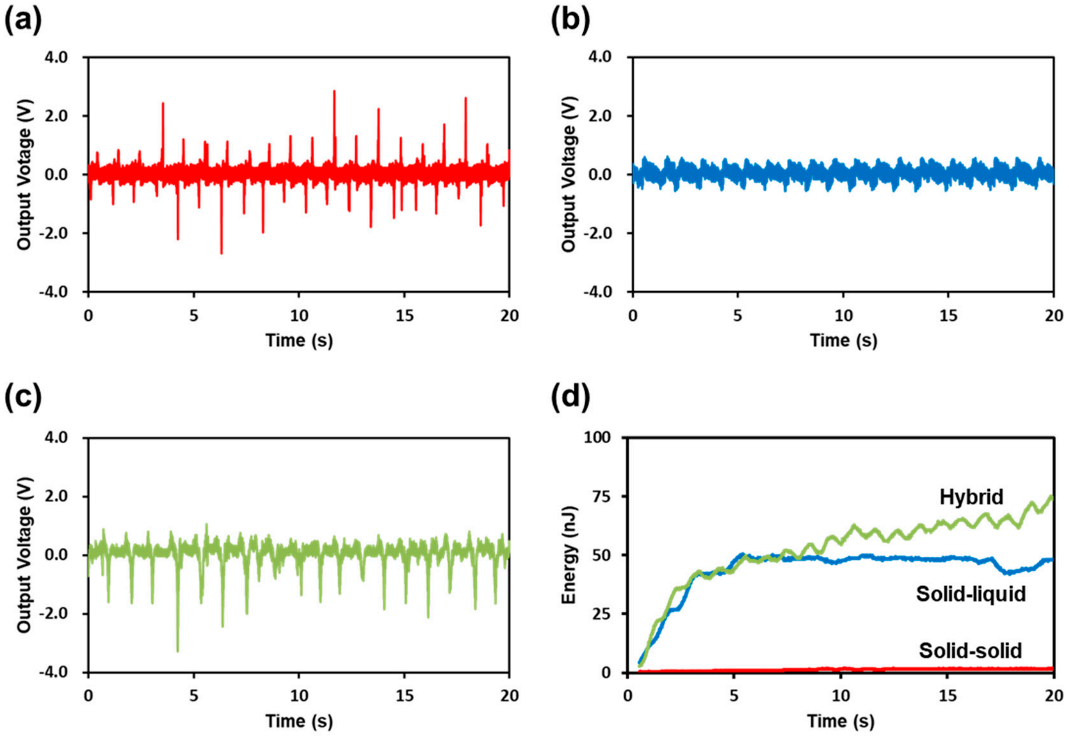

Figure S3 Electrical output of the TENG under wave motion with a frequency of 1 Hz in (a) solid-solid, (b) solid-liquid, and (c) hybrid triboelectrification. (d) Charged energy of the 1 μF capacitor with a 1 Hz wave frequency
